# Research progress on HPV-related vaginal wall lesions after hysterectomy

**DOI:** 10.3389/fonc.2026.1897256

**Published:** 2026-08-20

**Authors:** Dong Zhang, Boqun Xu

**Affiliations:** Department of Obstetrics and Gynecology, Women’s Hospital of Nanjing Medical University / Nanjing Women and Children’s Healthcare Hospital, Nanjing, China

**Keywords:** human papillomavirus infection, hysterectomy, persistent infection, precancerous lesion, vaginal intraepithelial neoplasia

## Abstract

Hysterectomy is one of the most commonly performed gynecological surgeries worldwide. Vaginal wall lesions after hysterectomy, particularly vaginal intraepithelial neoplasia (VaIN) associated with High-risk human papillomavirus (HR-HPV) infection, have become a focal point in gynecological oncology research. With the widespread implementation of cervical cancer screening programs and the increasing awareness of HPV-related diseases, the detection rate of VaIN has gradually increased. This review systematically analyzes the research progress in various aspects of HPV-related vaginal wall lesions after hysterectomy, including epidemiological characteristics, risk factors, pathogenesis, clinical features, diagnostic strategies, treatment modalities, and follow-up management. Epidemiological studies have shown that although VaIN accounts for less than 1% of all lower genital tract precancerous lesions, its incidence is significantly higher in patients who have undergone hysterectomy for cervical intraepithelial neoplasia (CIN) or cervical cancer. The main risk factors include age, menopausal status, preoperative high-grade CIN, high-risk HPV infection (especially HPV16 and HPV18), immunosuppressive status, surgical approach, positive surgical margins, smoking, multiple sexual partners, and vaginal microecological disorders. The pathogenesis of VaIN after hysterectomy is complex and multifactorial. Persistent HPV infection plays a central role. The E6 and E7 oncoproteins of high-risk HPV promote malignant transformation by degrading p53 and inactivating retinoblastoma protein (pRb), leading to uncontrolled cell proliferation. Surgery-induced anatomical changes, local immune microenvironment alterations, and latent HPV reactivation collectively contribute to VaIN development. Diagnosis requires a standardized approach combining HPV testing, liquid-based cytology (TCT), colposcopy, and targeted biopsy. Colposcopic examination should focus on the vaginal apex, suture line, and lateral fornices where lesions commonly hide. Treatment should be individualized based on lesion grade, extent, location, patient age, fertility requirements, and previous treatments. Options include surgical excision (partial vaginectomy, vaginal apex resection), ablative therapy (CO_2_ laser), topical medications (imiquimod), photodynamic therapy (PDT), and radiotherapy for invasive cancer. Despite effective initial treatment, VaIN has a high recurrence rate, with cumulative recurrence reaching up to 80% within 2–5 years. Long-term follow-up with risk-stratified surveillance is essential. Annual HPV testing combined with TCT is recommended for high-risk patients. Artificial intelligence-assisted colposcopy and molecular biomarkers represent promising future directions. This review provides a theoretical basis for standardized diagnosis and treatment of VaIN after hysterectomy and identifies current challenges and controversies in clinical practice.

## 1 Introduction

Vaginal intraepithelial neoplasia (VaIN) accounts for approximately 0.4% of all lower genital tract epithelial lesions in women. Due to its relatively low incidence, VaIN has received limited clinical attention in the past. However, with the widespread implementation of cervical cancer and precancerous lesion screening in recent years, a considerable number of patients with cervical high-grade squamous intraepithelial lesion (HSIL), adenocarcinoma *in situ* (AIS), or early-stage cervical cancer have undergone hysterectomy (1). Surgery is not a once-and-for-all solution, as the risk of lesions at the vaginal vault and vaginal wall persists postoperatively. Among these, VaIN is the most common and is closely associated with persistent human papillomavirus (HPV) infection. Although the overall incidence of VaIN—a precursor to vaginal invasive cancer—remains low, it increases significantly among patients who have undergone hysterectomy for cervical lesions, becoming an important issue affecting long-term quality of life and psychological well-being. Post-hysterectomy alterations in vaginal anatomy, changes in the immune microenvironment, and persistent HPV infection collectively form a complex backdrop for the development and progression of VaIN. Currently, there is no unified consensus regarding the pathogenesis, optimal diagnostic methods, treatment strategies, or long-term follow-up protocols for postoperative VaIN, leading to variations in clinical practice. This review synthesizes recent research advances on HPV-related vaginal lesions following hysterectomy, aiming to provide a reference for clinical management.

## 2 Epidemiology and risk factors

### 2.1 Epidemiological features

VaIN accounts for less than 1% of all lower genital tract precancerous lesions, with an annual incidence of approximately 0.2–2 per 100,000 women in the general population. However, among patients who undergo hysterectomy for cervical intraepithelial neoplasia (CIN) or cervical cancer, the incidence of postoperative VaIN is significantly higher. A systematic review and meta-analysis by Li et al. (2026) (2), including 48 studies with 18,959 patients, reported an overall incidence of VaIN 1+ of 2.7% (95% CI: 1.8%–3.7%) following hysterectomy for CIN or cervical cancer, with an incidence of vaginal cancer of 0.3‰. However, several methodological limitations warrant consideration when interpreting these findings. First, substantial heterogeneity (I² > 70%) was observed across the included studies, likely attributable to variations in screening protocols, HPV testing methods, follow-up durations, and geographic differences in HPV genotype distribution. Second, publication bias cannot be excluded, as studies with higher incidence rates may be more likely to be published. Third, the majority of included studies were retrospective in design, with inherent risks of selection bias and incomplete data collection. Fourth, definitions of VaIN and the thresholds for biopsy confirmation varied considerably across studies. Despite these limitations, the meta-analysis provides the most comprehensive estimate currently available and clearly demonstrates the elevated risk in this specific patient population. The study also found an increasing trend over time: the incidence was 1.7% in studies published before 2000, rising to 4.7% in later studies, indicating a growing clinical concern. Notably, the detection rate of VaIN is markedly higher among patients who undergo hysterectomy for HSIL, AIS, or cervical cancer. Kong (2021) (3) reported a higher incidence of VaIN II+ following hysterectomy for CIN II or worse. Another study by Ma (2020) (4), which included 174 patients undergoing hysterectomy, found a higher VaIN incidence among those with a history of CIN compared to those without. Wang (2023) (5)demonstrated that most postoperative VaIN occurs within five years after hysterectomy, predominantly in the upper one-third of the vagina, particularly at the vaginal vault suture line and bilateral fornices, and is often multifocal, posing challenges for early detection and comprehensive assessment.

### 2.2 Risk factors

The development of VaIN after hysterectomy results from the interplay of multiple factors, which can be categorized into patient-related, surgery-related, and other environmental/behavioral factors.

Patient-related factors: Age and menopausal status are major determinants. Bruno et al. (2024) (6)reported that estrogen deficiency after menopause leads to thinning of the vaginal mucosa, reduced glycogen content, parakeratosis of superficial cells, and deepening of mucosal folds, which decrease the adequacy of colposcopy and may obscure or accentuate lesions. Postmenopausal vaginal epithelium exhibits reduced Langerhans cell density and lower levels of antimicrobial peptides in secretions, weakening local clearance of persistent HPV infection and creating conditions for viral persistence and reactivation. Preoperative cervical HSIL is a major risk factor for postoperative VaIN. High-risk HPV types, particularly HPV16 and HPV31, are associated with the development and progression of VaIN (7) and also with recurrence (8). Conflicting findings have emerged regarding the relative contribution of specific HPV genotypes. While some studies have identified HPV16 as the predominant genotype associated with high-grade VaIN and progression to invasive disease, others have reported a more balanced distribution of HPV16, HPV18, and HPV31. These discrepancies may be explained by geographic variations in HPV prevalence, differences in study populations (e.g., immunocompetent versus immunosuppressed patients), and the timing of HPV testing relative to lesion detection. Furthermore, the evidence for genotype-specific risk stratification in VaIN remains less robust than that established for cervical lesions, warranting caution in clinical decision-making based solely on HPV genotype. Immunosuppressive conditions, including post-transplantation status and HIV infection, are established risk factors (9).

Surgery-related factors: The surgical approach influences the incidence of postoperative VaIN. Wang (2025) (10) suggested that laparoscopic hysterectomy may be associated with higher tumor recurrence rates compared to open surgery; however, further evidence is needed to confirm the role of pneumoperitoneum and uterine manipulator use in tumor cell dissemination and HPV spread. Surgical margin status is critically important: positive margins indicate residual disease and are a major risk factor for postoperative recurrence.

Vaginal microbiota-related factors: A retrospective study (11) showed that among patients undergoing surgery for CIN or cervical cancer, 142 (68.6%) had postoperative vaginal microecological imbalance, with 125 (60.4%) exhibiting <50% *Lactobacillus* species and 105 (50.7%) having a vaginal pH ≥4.5. The study further demonstrated a significant association between microecological imbalance and persistent postoperative high-risk HPV infection (OR = 4.640, 95% CI: 2.085–10.460, *P* < 0.001). Cell-free supernatant of bacteria associated with bacterial vaginosis, particularly *Gardnerella* species, significantly inhibited the proliferation of vaginal fibroblasts and the production of type I collagen. Fibroblasts play a crucial role in tissue repair and maintenance of mucosal integrity. Following hysterectomy, the vaginal vault undergoes a healing and remodeling process. Dysbiosis-induced impairment of fibroblast function may lead to poor wound healing and persistent epithelial barrier defects, providing a pathological basis for persistent HPV infection and VaIN development. Notably, *Lactobacillus* supernatant did not exert such inhibitory effects on fibroblasts (12).

Other factors: Smoking, multiple sexual partners, multiple pregnancies, and other behavioral and lifestyle factors have been shown to increase the risk of VaIN.

## 3 Pathogenesis

Persistent HR-HPV infection is the underlying cause of VaIN following hysterectomy.

### 3.1 Impact of surgery on the local microenvironment

Total hysterectomy inevitably alters the normal morphology of the vagina. The closure of the vaginal vault creates a new squamocolumnar junction (transformation zone). Scar contracture leads to the formation of crypts at the bilateral fornices, and these structural changes disrupt the local mucosal immune barrier, creating conditions conducive to persistent HPV colonization and infection (13). A case report demonstrated that even with negative surgical margins, high-grade cytological abnormalities (ASC-H) and positive HPV testing can be detected at the vaginal vault as early as four months after total hysterectomy (14), directly confirming the association between persistent HPV infection at the altered vault and lesion recurrence. Furthermore, surgery itself induces changes in the vaginal microbiota and cytokine profiles, further influencing postoperative HPV clearance efficiency.

### 3.2 Persistent HPV infection

In patients undergoing hysterectomy for HPV-related diseases (e.g., CIN), latent HPV infection may persist in the vaginal mucosa or residual cervical tissue. Surgical stress and alterations in the local immune environment can reactivate these latent viruses, leading to renewed viral replication and expression of oncoproteins. Additionally, *de novo* HPV infection can occur postoperatively.

### 3.3 Viral oncogenic mechanisms

Qi (2023) (15) highlighted that the E6 and E7 oncoproteins of HR-HPV drive malignant cellular transformation. The E6 protein promotes the degradation of the p53 tumor suppressor protein, thereby inhibiting apoptosis. The E7 protein binds to retinoblastoma protein (pRb), leading to its inactivation and subsequent release of cell cycle inhibition, which results in excessive cellular proliferation. Following integration of viral DNA into the host genome, the oncogenic effects become more persistent and stable, ultimately driving the progression from low-grade VaIN to high-grade VaIN and even invasive carcinoma.

### 3.4 Emerging molecular biomarkers

Beyond HPV DNA testing, emerging molecular biomarkers show promise for improving risk stratification and predicting disease progression in post-hysterectomy VaIN. HPV E6/E7 mRNA detection (16), which reflects active viral oncogene transcription, has demonstrated higher specificity for clinically significant lesions compared to HPV DNA testing alone. A positive E6/E7 mRNA result indicates transcriptionally active HPV infection and may better identify patients at true risk of progression. Host DNA methylation markers (17, 18), including CADM1, PAX1, MAL, and FAM19A4, have shown potential in detecting high-grade lesions across the lower genital tract. Methylation of these tumor suppressor genes is an early event in HPV-driven carcinogenesis and may precede morphological changes detectable by colposcopy. p16/Ki-67 dual immunocytochemistry (16) provides simultaneous detection of cell cycle dysregulation (p16 overexpression) and proliferation (Ki-67), offering a molecular surrogate for transforming HPV infection. While these biomarkers have been extensively studied in cervical lesions, their specific application to vaginal lesions after hysterectomy remains limited. Host immune response markers, such as circulating HPV-specific T-cell responses and vaginal cytokine profiles, represent additional promising avenues for predicting HPV clearance versus persistence. However, prospective validation in large cohorts of post-hysterectomy patients is needed before these biomarkers can be incorporated into routine clinical practice.

## 4 Clinical features and diagnostic approaches

### 4.1 Clinical features

The diagnosis of VaIN after hysterectomy should be based on a standardized evaluation process, integrating patient history and ancillary tests. Because postoperative VaIN may result from multiple factors—including pre-existing HPV infection, occult lesions at the vaginal vault or wall undetected during surgery, or lesion progression due to irregular postoperative follow-up—a comprehensive analysis is essential to avoid oversimplification of the underlying mechanism (19).

### 4.2 Diagnostic methods and strategies

For patients after hysterectomy, particularly those who underwent surgery for CIN or cervical cancer, regular combined screening with HPV testing and ThinPrep cytology test (TCT) is recommended. HPV testing offers high sensitivity for detecting HR-HPV infection. TCT, reported using the TBS system (20), defines abnormalities as atypical squamous cells of undetermined significance (ASC-US) or worse. A retrospective study of 168 VaIN patients reported an overall sensitivity of 92.4% for HPV testing alone, significantly higher than the 61.5% sensitivity of cytology alone (*P* = 0.001); the combination of cytology and HPV testing increased sensitivity to 95.0%, indicating that combined testing improves lesion detection (21).

Colposcopy with targeted biopsy (22) is the cornerstone for confirming VaIN. Colposcopy is essential for diagnosis, with particular attention to the upper vagina, vaginal vault, and lateral fornices, where lesions may be hidden. For postmenopausal patients, short-term preoperative use of topical vaginal estrogen cream can improve the vaginal mucosal condition and enhance colposcopic visibility. Colposcopic features of VaIN (23) typically include acetowhite epithelium, punctation, mosaicism, or iodine-negative areas. Given the often multifocal distribution of VaIN, multiple biopsies of the vaginal vault, bilateral fornices, and other suspicious areas are recommended to improve diagnostic accuracy. To facilitate long-term postoperative follow-up, techniques such as transvaginal closure of the vaginal vault during surgery may reduce the formation of fornix crypts and lower the difficulty of future examinations (24).

Imaging studies are valuable in the evaluation of high-grade VaIN or suspected invasive carcinoma, serving three main roles: (1) assessing the presence and local extent of invasion; (2) providing anatomical guidance for treatment selection; and (3) monitoring for recurrence or progression during post-treatment follow-up. Pelvic contrast-enhanced MRI is the preferred modality for evaluating the depth and extent of local invasion in vaginal lesions. If distant metastasis is suspected, whole-body FDG-PET-CT or contrast-enhanced CT of the chest, abdomen, and pelvis is recommended for comprehensive disease assessment.

Artificial intelligence (AI)-assisted image analysis (25) (26), are poised to transform colposcopic evaluation. Deep learning algorithms trained on large datasets of colposcopic images have demonstrated promising performance in identifying acetowhite epithelium and vascular patterns characteristic of high-grade lesions. Preliminary studies suggest that AI-assisted colposcopy may improve sensitivity for detecting small or subtle lesions, reduce inter-observer variability, and potentially guide biopsy site selection. However, current AI models face several limitations: (1) they require large, well-annotated training datasets that are often unavailable for rare conditions like VaIN; (2) performance may be compromised by variations in image acquisition protocols and colposcope types; and (3) prospective validation in diverse clinical settings is still lacking. Integration of AI with other clinical and molecular data (e.g., HPV genotype, methylation markers) may ultimately enable more personalized risk stratification and surveillance strategies.

## 5 Treatment strategies for vaginal wall lesions after hysterectomy

The treatment of vaginal wall lesions following hysterectomy should be individualized, taking into account the lesion grade, extent, location, patient age, prior treatment history, and overall systemic condition. The therapeutic goals are to eliminate the lesion, prevent malignant transformation, and preserve vaginal function to the greatest extent possible. The overall treatment pathway includes surgical and non-surgical approaches, and the choice of therapy should be made after rigorously excluding invasive carcinoma, based on evidence-based medicine and patient preferences.

In 2023, the European Society of Gynaecological Oncology (ESGO), the International Society for the Study of Vulvovaginal Disease (ISSVD), the European Federation for Colposcopy (EFC), and the European College for the Study of Vulvovaginal Disease (ECSVD) jointly issued an international consensus on the management of VaIN (27). This consensus proposes a stratified management approach based on lesion grade: VaIN I (low-grade vaginal SIL) may be managed by observation, whereas VaIN 2–3 (high-grade vaginal SIL) should be treated. Treatment options should be individualized according to patient characteristics, lesion extent, and prior treatment history.

### 5.1 Surgical treatment

Local excisional surgery (partial vaginectomy, focal lesion excision) is indicated for VaIN 2–3 with localized, well-defined lesions. Under colposcopic guidance, excision should extend 2–3 mm beyond the lesion margin and reach the fascial layer to achieve negative margins. This approach provides a complete pathological specimen to confirm the absence of invasion and offers a high cure rate.

Transvaginal vaginectomy of the vaginal vault is suitable for VaIN 2–3 confined to or predominantly involving the vaginal vault. Indermaur (2005) (28) reported that this transvaginal approach, involving circumferential excision of the vaginal vault and a portion of the upper vaginal wall, can completely remove lesions within crypts. Studies have demonstrated a high cure rate and low surgery-related complication rate, making it an effective treatment for high-grade VaIN at the vault.

### 5.2 Non-surgical treatment

Ablative therapy: Carbon dioxide (CO_2_) laser ablation is a commonly used method for multifocal, superficial VaIN 2–3. Under colposcopic guidance, the lesional epithelium is vaporized with a laser, typically to a depth of 1–2 mm, destroying the full thickness of the epithelium without damaging the underlying stroma. Advantages include precise control of the ablation range, rapid healing, and minimal impact on vaginal elasticity and function. Wang (2022) (29) reported a lesion regression rate exceeding 75%. The main disadvantage is the inability to obtain a histopathological specimen, with a risk of incomplete treatment, necessitating rigorous exclusion of invasive carcinoma.

Topical pharmacotherapy: Immune modulators (e.g., imiquimod) act by inducing local cytokine production, thereby activating cellular immunity to clear HPV infection and abnormal cells (30). A 5% cream formulation is commonly used, typically applied intravaginally 1–3 times per week. Studies have shown that this regimen has certain efficacy for high-grade VaIN, providing an alternative for patients who are unsuitable for or unwilling to undergo surgery. The main adverse effects are local irritative symptoms, which are generally tolerable. It should be noted that the approved indication for imiquimod is condyloma acuminatum; its use for VaIN is off-label and requires fully informed consent. Regarding topical pharmacotherapy, the ESGO and other societies’ consensus explicitly states that imiquimod is associated with the lowest recurrence rate and the highest HPV clearance rate, and may be considered the optimal topical treatment option. In contrast, trichloroacetic acid and 5-fluorouracil are considered historical regimens and should no longer be recommended. For VaIN occurring after hysterectomy for CIN 3, the consensus specifically emphasizes that laser vaporization and topical pharmacotherapy are not optimal because they cannot reach the epithelium buried within vaginal scar tissue; in such cases, surgical treatment should be prioritized (27).

A critical appraisal of the evidence base for these treatment recommendations reveals several important limitations (31). First, the majority of studies on CO_2_ laser ablation and topical pharmacotherapy are retrospective case series with small sample sizes, lacking control groups and standardized outcome definitions. The reported cure rates of 70–85% may overestimate true efficacy due to selection bias and publication bias. Second, only a few prospective randomized controlled trials have directly compared different treatment modalities, and most have been underpowered to detect meaningful differences in recurrence rates. Third, the evidence for treatment effectiveness specifically in the post-hysterectomy population is particularly sparse, as many studies have included mixed populations of patients with and without prior hysterectomy. Fourth, the optimal treatment for multifocal or recurrent high-grade VaIN remains undefined, with no clear consensus on whether repeated local treatments or more radical approaches (e.g., total vaginectomy) are preferred. These evidence gaps underscore the need for well-designed comparative effectiveness studies in this specific patient population.

Photodynamic therapy (PDT): A photosensitizer such as 5-aminolevulinic acid is applied to the lesion. This agent is selectively taken up by abnormally proliferating and metabolically active cells, and subsequent irradiation with red light of a specific wavelength generates singlet oxygen, which selectively destroys lesional cells. A study by Yao H (2025) (32) demonstrated that ALA-PDT achieved a higher complete remission rate for VaIN compared with CO_2_ laser, along with a higher HPV clearance rate, fewer adverse effects, less damage to normal tissue, and a clear advantage in preserving vaginal anatomy and function. However, the treatment is costly and technically complex.

Radiotherapy is an important radical treatment modality for invasive carcinoma of the vaginal vault. It typically involves external beam radiotherapy combined with brachytherapy. Ayijiamali Aihemaiti et al. (2023) (33) noted that high-dose-rate intracavitary brachytherapy (HDR-ICBT) can be considered for extensive, multifocal, or recurrent high-grade VaIN, particularly in elderly patients who cannot tolerate surgery or in whom other treatments have repeatedly failed. Studies have shown a high control rate; however, attention must be paid to long-term complications, including vaginal atrophy, stenosis, radiation proctitis, and radiation cystitis.

## 6 Recurrence risk and follow-up management strategies

The biological behavior of vaginal intraepithelial neoplasia shares similarities with that of cervical intraepithelial neoplasia, namely the possibility of spontaneous regression. Existing evidence indicates that the lower the lesion grade, the higher the likelihood of spontaneous regression. Although most patients with VaIN II–III achieve clinical cure after standardized treatment, a subset remains at risk of recurrence or even progression to invasive vaginal carcinoma. These observations underscore the critical importance of establishing a long-term follow-up system for these patients.

### 6.1 Recurrence rate and risk factors

Reported recurrence rates for VaIN after treatment vary across studies due to differences in sample size, treatment modality, and follow-up duration. Kechagias KS et al. (2022) (34) noted that the cumulative recurrence rate for high-grade VaIN at 2 to 5 years after treatment can be as high as 80%. However, this striking figure warrants careful interpretation. The estimate was derived from a meta-analysis that included studies with heterogeneous definitions of recurrence (e.g., histological confirmation versus cytological abnormality alone), varying follow-up protocols, and different treatment modalities. The high recurrence rate may partially reflect persistent HPV infection rather than true disease recurrence, as HPV positivity without histological lesions is often included in composite recurrence outcomes. Additionally, the included studies had high rates of loss to follow-up, which may have introduced bias. Nevertheless, even when more stringent definitions of histologically confirmed recurrence are applied, the recurrence rate remains substantial, supporting the clinical importance of rigorous surveillance. This finding clearly demonstrates that for VaIN occurring after hysterectomy, even after effective initial treatment, the risk of recurrence and progression remains substantial. Studies indicate that recurrence risk is not uniformly distributed; the following populations are at particularly high risk: (1) patients with a pathological diagnosis of VaIN III; (2) patients previously treated for CIN or cervical cancer; and (3) patients with initial cervical cancer. A comprehensive analysis by Li Xing et al. (2022) (35) also identified several factors contributing to recurrence: lesion characteristics (multifocal lesions, due to their wide extent and indistinct borders, are prone to residual disease after treatment); infection status (persistent postoperative HR-HPV infection is one of the strongest predictors of recurrence); and patient-related factors (immunosuppression, such as after organ transplantation or HIV infection, and long-term smoking history have been shown to significantly increase recurrence risk).

### 6.2 Follow-up strategies and implementation protocols

Given the risk of recurrence, both domestic and international consensus strongly advocates for rigorous, long-term follow-up for patients with VaIN, particularly those who have undergone hysterectomy. According to Chen Y (2023) (36), the primary goal of follow-up is early detection of residual, recurrent, or progressive lesions to enable timely intervention. Currently recommended follow-up protocols generally adopt a risk-stratified management approach. The ESGO and other societies’ consensus provides clear recommendations for follow-up: the first post-treatment evaluation should be performed at 6 months, including cytology and HPV testing; thereafter, follow-up should be conducted every 6 months for 2 years, then annually thereafter (27). Despite these consensus recommendations, significant controversy remains regarding the optimal intensity and duration of follow-up (37). Proponents of intensive surveillance argue that the high recurrence rate (up to 80% by 5 years) justifies frequent testing to enable early intervention and prevent progression to invasive carcinoma. Conversely, critics contend that the psychological burden, healthcare costs, and potential harms of unnecessary interventions from false-positive results may outweigh the benefits of very frequent surveillance, particularly for low-risk patients. The cost-effectiveness of different follow-up strategies has not been rigorously evaluated in the post-hysterectomy VaIN population. Furthermore, the optimal duration of follow-up remains undefined; some experts advocate for lifelong surveillance, while others suggest that patients with sustained HPV clearance and normal cytology for 5 years may be transitioned to less frequent screening. Individualized, risk-stratified approaches that incorporate HPV genotype, lesion grade, treatment history, and host immune status may offer a rational middle ground, but prospective validation of such risk models is needed. The consensus also emphasizes that VaIN has a high propensity for recurrence, making patient adherence to close follow-up essential, and that the impact of treatment on quality of life (including psychological and psychosexual issues) should be addressed and managed. Perkins et al (2021) (38) proposed that the initial intensive follow-up period should include a comprehensive evaluation at 6 months after treatment completion. This evaluation should include ThinPrep cytology test (TCT) and high-risk HPV (HR-HPV) testing. Negative results for both indicate effective treatment and viral clearance, but surveillance should not be relaxed.

The intermediate follow-up period extends to 2 years after treatment, during which HPV testing is the primary monitoring modality, as HPV clearance is a key indicator of long-term recurrence risk. During this phase, HPV testing should be performed every 6 to 12 months, with TCT added as needed.

Long-term annual follow-up: After 2 years of follow-up, if HPV testing is negative and cytology is normal, patients may transition to annual routine screening. However, for patients who initially had high-grade lesions (VaIN II–III), multifocal lesions, or concomitant other high-risk factors (e.g., immunosuppression, history of persistent HPV infection), a higher intensity of monitoring and longer follow-up duration should be maintained even after entering the long-term follow-up phase. According to Zhu XH (2021) (39), annual follow-up should include at least combined TCT and HR-HPV testing.

Role of colposcopy: Colposcopy should not be performed as a routine component of every follow-up visit, but it plays an irreplaceable role in specific circumstances. Colposcopy with targeted biopsy is mandatory in the following situations: (1) TCT results indicating atypical squamous cells of undetermined significance (ASC-US) or higher, particularly ASC-H or HSIL; (2) persistent positive HR-HPV testing, especially for high-risk types HPV16 and HPV18; and (3) clinical examination or patient-reported symptoms suggestive of abnormality, such as a suspicious neoplasm or unexplained bleeding.

### 6.3 Special considerations in follow-up

Based on current clinical research and practical experience, for postmenopausal patients or those with vaginal atrophy or stenosis after hysterectomy that makes examination difficult, short-term use of topical estrogen cream (typically for 2–4 weeks) before cytological sampling or colposcopy is recommended. This pretreatment improves vaginal mucosal thickness, elasticity, and blood supply, significantly enhancing the representativeness of cytology samples and the clarity and adequacy of colposcopic visualization, thereby improving the accuracy of detecting lesions at the vaginal vault and walls.

In addition to medical techniques, successful long-term follow-up management requires systematic patient education. Shen Yingli (2025) (40)emphasized that healthcare providers should explain to patients the necessity of adhering to regular, long-term follow-up and clarify the relationship between persistent HPV infection and lesion recurrence or progression, in order to improve patient compliance with follow-up.

## 7 HPV vaccination: current evidence and future directions

Prophylactic HPV vaccination has dramatically reduced the incidence of cervical cancer and high-grade cervical lesions in vaccinated cohorts. However, its role in preventing VaIN after hysterectomy is less well-established. The current evidence base includes: (1) ecological studies demonstrating reductions in genital warts and low-grade vaginal lesions in vaccinated populations; (2) *post-hoc* analyses of vaccine trials suggesting potential protection against vaginal lesions; and (3) observational studies reporting lower rates of HPV-related vaginal disease in vaccinated women.

For patients who have already undergone hysterectomy for HPV-related cervical disease, several unresolved questions remain: (1) Does HPV vaccination after hysterectomy reduce the risk of subsequent VaIN? (2) Can vaccination promote clearance of persistent postoperative HPV infection? (3) What is the cost-effectiveness of perioperative vaccination in this specific population?

Limited evidence suggests that (41) post-treatment vaccination may be associated with reduced recurrence rates of HPV-related lesions. A meta-analysis by Kechagias et al. (2022) (34) found that HPV vaccination after surgical treatment for CIN was associated with a lower risk of recurrence, and this finding may plausibly extend to VaIN. However, this evidence comes from secondary analyses and observational studies, with inherent confounding and indication bias. Direct evidence specifically addressing VaIN after hysterectomy is virtually non-existent. The biological plausibility for a therapeutic or secondary preventive effect of vaccination after hysterectomy is supported by the potential for vaccine-induced antibodies to neutralize HPV in the vaginal mucosa and residual tissue, reducing the viral reservoir and preventing reinfection.

Until direct evidence emerges, clinical decisions regarding HPV vaccination in this population should be individualized, weighing potential benefits against costs. The ESGO consensus (27) does not provide specific recommendations for or against vaccination after hysterectomy, reflecting the current evidence gap. Future research should prioritize randomized controlled trials of HPV vaccination in women undergoing hysterectomy for CIN or cervical cancer, with long-term outcomes including VaIN incidence, HPV clearance rates, and recurrence risk.

## 8 Current controversies and unresolved issues

Despite significant advances in our understanding of VaIN after hysterectomy, several controversies and unresolved issues persist in clinical practice.

Controversy 1: Optimal surgical approach. Whether minimally invasive hysterectomy is associated with a higher risk of postoperative VaIN compared to open surgery remains debated. Wang et al. (2025) (10) suggested a potential association with laparoscopic approaches, but the evidence is confounded by differences in patient populations, surgical techniques, and follow-up protocols. But they did not specifically examine VaIN as an outcome, and no dedicated studies have directly compared VaIN incidence by surgical approach with adequate adjustment for confounding. The mechanism by which minimally invasive surgery might increase risk is speculative at best.

Controversy 2: Management of VaIN I. The 2023 ESGO consensus (27) recommends observation for VaIN I, but the optimal observation strategy is unclear. While the majority of VaIN I lesions regress spontaneously, a small proportion progress to high-grade disease. Identifying which patients warrant closer surveillance versus reassurance remains challenging, as no validated predictive biomarkers exist for VaIN I progression. The natural history of VaIN I specifically in the post-hysterectomy population has not been well-characterized.

Controversy 3: Follow-up intensity and duration. As discussed in Section 6, the optimal follow-up schedule remains undefined. The tension between early detection of recurrence and the psychological/financial costs of surveillance has not been resolved. Risk-stratified approaches have been proposed but lack prospective validation.

Controversy 4: Treatment of recurrent disease. The optimal management of recurrent VaIN after initial treatment is particularly challenging. Cumulative evidence suggests that repeated local excisions or ablations may lead to vaginal shortening, scarring, and stenosis, compromising quality of life. The threshold for proceeding to more radical surgery (e.g., total vaginectomy) or radiotherapy is undefined and varies considerably across centers. Individualized decision-making integrating patient preferences, lesion characteristics, and prior treatment history is essential but lacks a clear evidence-based framework.

## 9 Conclusion and future perspectives

HPV-related vaginal wall lesions after hysterectomy, particularly VaIN, represent a long-term complication that warrants high clinical attention. The development of these lesions is associated with the patient’s prior HPV infection status, surgical factors, and postoperative alterations in the local immune microenvironment. Zhou Yurong (2024) (42)noted that the current combination of HPV/TCT co-testing, meticulous colposcopic examination, and biopsy enables early diagnosis.

However, challenges remain in this field. There is no gold standard for the optimal treatment of multifocal or recurrent high-grade VaIN (Zhuang Xinrong, 2023) (43). The long-term efficacy and safety of emerging therapies—such as photodynamic therapy and immunomodulators—require confirmation through larger, prospective studies. Strategies for postoperative follow-up that balance screening benefits with healthcare costs still need to be explored. Furthermore, the long-term impact of different surgical approaches (minimally invasive vs. open surgery) on the incidence of postoperative HPV-related vaginal lesions requires higher-level evidence-based data.

Future research directions include:

1. Large-scale prospective cohort studies. Dedicated prospective studies with standardized case definitions, uniform follow-up protocols, and comprehensive data collection (including HPV genotype, treatment history, and immune status) are urgently needed to define the true incidence, natural history, and risk factors for VaIN after hysterectomy.
2. Comparative effectiveness trials. Multicenter randomized controlled trials comparing treatment modalities with clinically meaningful outcomes (durable response, recurrence-free survival, quality of life) would resolve many current uncertainties. Pragmatic trials that reflect real-world clinical decision-making are particularly valuable.
3. Immunological and microbiome research. Elucidating the mechanisms by which hysterectomy alters the local immune microenvironment and vaginal microbiota may identify novel targets for prevention and therapy. The relationship between specific bacterial species, immune cell populations, and HPV persistence warrants further investigation.
4. Biomarker development and validation. High-quality studies validating molecular biomarkers (methylation markers, E6/E7 mRNA, p16/Ki-67) specifically in the post-hysterectomy VaIN population are needed. Biomarkers that predict progression from VaIN I to VaIN II–III and recurrence after treatment would transform risk stratification.
5. Integration of artificial intelligence. AI-assisted colposcopy with deep learning algorithms holds promise for improving lesion detection and guiding biopsy. Integration of AI with clinical and molecular data may enable personalized risk prediction. Prospective validation in diverse clinical settings is essential.
6. Therapeutic vaccines and immunotherapies. The development of therapeutic HPV vaccines and immune checkpoint inhibitors for HPV-related lower genital tract diseases is advancing rapidly. While these approaches have shown promise in cervical cancer, their role in VaIN, particularly in the post-hysterectomy setting, remains to be explored.
7. HPV vaccination research. As discussed in Section 7, randomized controlled trials of HPV vaccination in women undergoing hysterectomy for CIN or cervical cancer are a priority. These studies should include VaIN incidence as a key secondary outcome and assess cost-effectiveness.

Ultimately, a multidisciplinary approach integrating gynecologic oncology, virology, immunology, and health services research will be required to optimize outcomes for patients with HPV-related vaginal lesions after hysterectomy.
